# Clonal Genotype of *Geomyces destructans* among Bats with White Nose Syndrome, New York, USA

**DOI:** 10.3201/eid1707.102056

**Published:** 2011-07

**Authors:** Sunanda S. Rajkumar, Xiaojiang Li, Robert J. Rudd, Joseph C. Okoniewski, Jianping Xu, Sudha Chaturvedi, Vishnu Chaturvedi

**Affiliations:** Author affiliations: New York State Department of Health, Albany, New York, USA (S.S. Rajkumar, X. Li, R.J. Rudd, S. Chaturvedi, V. Chaturvedi);; New York State Department of Environmental Conservation, Albany (J.C. Okoniewski);; McMaster University, Hamilton, Ontario, Canada (J. Xu);; State University of New York at Albany, Albany (S. Chaturvedi, V. Chaturvedi)

**Keywords:** fungi, fungal infection, bats, Geomyces destructans, white nose syndrome, population biology, New York, dispatch

## Abstract

The dispersal mechanism of *Geomyces destructans*, which causes geomycosis (white nose syndrome) in hibernating bats, remains unknown. Multiple gene genealogic analyses were conducted on 16 fungal isolates from diverse sites in New York State during 2008–2010. The results are consistent with the clonal dispersal of a single *G. destructans* genotype.

Geomycosis, or white nose syndrome, is a newly recognized fungal infection of hibernating bats. The etiologic agent, the psychrophilic fungus *Geomyces destructans*, was first recognized in caves and mines around Albany, New York, USA (1,2). The disease has spread rapidly in New York and other states in the northeastern United States. At least 1 affected bat species is predicted to face regional extinction in the near future (3). Much remains unknown about this fungus, including its ecology and geographic distribution. For example, although hibernacula are high on the list of suspected sites, where the bats acquire this infection is not known. Similarly, although strongly suspected, the role of humans and other animals in the dispersal of *G. destructans* and the effect of such dispersals in bat infections have not been confirmed. We recently showed that 6 *G. destructans* strains from sites near Albany were genetically similar (2), raising the possibility of a common source for the spread of this infection. Corollary to this observation and other opinions (3,4), the US Fish & Wildlife Service has made an administrative decision to bar human access to caves as a precautionary measure (www.fws.gov/whitenosesyndrome/pdf/NWRS_WNS_Guidance_Final1.pdf). Thus, an understanding of the dispersal mechanism of *G. destructans* is urgently needed to formulate effective strategies to control bat geomycosis.

## The Study

We applied multiple gene genealogic analyses in studying *G. destructans* isolates; this approach yields robust results that are easily reproduced by other laboratories (5). Sixteen *G. destructans* isolates recovered from infected bats during 2008–2010 were analyzed. These isolates originated from 7 counties in New York and an adjoining county in Vermont, all within a 500-mile radius (Table 1). The details of isolation and identification of *G. destructans* from bat samples have been described (2). One isolate of a closely related fungus *G. pannorum* M1372 (University of Alberta Mold Herbarium, Edmonton, Alberta, Canada) was included as a reference control. To generate molecular markers, 1 isolate, *G. destructans* (M1379), was grown in yeast extract peptone dextrose broth at 15°C, and high molecular weight genomic DNA was prepared according to Moller et al. (6). A cosmid DNA library was constructed by using pWEB kit (Epicenter Biotechnologies, Madison, WI, USA) by following protocols described elsewhere (7). One hundred cosmid clones, each with ≈40-Kb DNA insert, were partially sequenced in both directions by using primers M13 and T7. The nucleotide sequences were assembled with Sequencher 4.6 (Gene Codes Corp., Ann Arbor, MI, USA) and BLAST (www.ncbi.nlm.nih.gov/BLAST) homology searches identified 37 putative genes. Sequences of 10 genes, including open reading frames, 3′ and/or 5′ untranslated regions, and introns, were evaluated as potential markers for analyzing *G. pannorum* and *G. destructans.* Our screening approach indicated that 8 gene targets could be amplified from both *G. destructans* and *G. pannorum* by PCR (Table 2).

**Table 1:** *Geomyces destructans* isolates studied, New York, USA

Isolate	Date obtained	Site, county*
M1379†	2008 Mar 28	Williams Hotel Mine, Ulster
M1380†	2008 Mar 28	Williams Hotel Mine, Ulster
M1381†	2008 Mar 28	Williams Hotel Mine, Ulster
M1383†	2008 Apr 11	Graphite Mine, Warren
M2325	2010 Jan 25	Westchester
M2327	2010 Feb 2	Dewitt, Onondaga
M2330	2009 Mar 5	Lancaster, Erie
M2331	2009 Mar 9	White Plains, Westchester
M2332	2009 Mar 11	Dannemora, Clinton
M2333	2009 Mar 11	Dannemora, Clinton
M2334	2009 Mar 12	Newstead, Erie
M2335	2009 Mar 16	Ithaca, Tompkins
M2336	2009 Oct 6	Bridgewater Mine, Windsor, VT
M2337	2010 Feb 9	Akron Mine, Erie
M2338	2010 Mar 4	Hailes Cave, Albany
M2339	2010 Mar 11	Letchworth Tunnel, Livingston


*All locations in New York state except Bridgewater Mine, Windsor, Vermont. †Previously analyzed by randomly amplified polymorphic DNA typing.

**Table 2:** *Geomyces destructans* and *G. pannorum* target gene fragments used for multiple gene genealogic analyses, New York, USA

Gene*	Homology (GenBank accession no.)	Amplicon size/ sequence used for comparison, bp	Primer sequence, 5′ → 3′†	*G. destructans*/*G. pannorum* GenBank accession nos.
*ALR*	*Penicillium marneffei* (XP_002152078.1)	654/534	V1905 (f): CGGAGTGAGATTTATGACGGC	HQ834314–HQ834329/HQ834330
			V1904 (r): CGTCCATCCCAGACGTTCATC	
*Bpntase*	*Glomerella graminicola* (EFQ33509.1)	921/745	V1869 (f): TCAGACGGACTCGGAGGGCAAG	HQ834331–HQ834346/HQ834347
			V1926 (r): TCGGTTACAGAGCCTCAGTCG	
*DHC1*	*Sordaria macrospora* (CBI53717.1)	597/418	V1906 (f): GGATGATTCGGTCACCAAACAG	HQ834348–HQ834363/HQ834364
			V1907 (r): ACAGCAAACACAGCGCTGCAAG	
*GPHN*	*Ajellomyces capsulatus* (EEH06836.1)	659/525	V1918 (f): CACTATTACATCGCCAGGCTC	HQ834365–HQ834380/HQ834381
			V1919 (r): CTAAACGCAGGCACTGCCTC	
*PCS*	*A. capsulatus* (EEH08767.1)	920/749	V1929 (f): AGGCTGCGATTGCTGAGTGC	HQ834382–HQ834397/HQ834398
			V1873 (r): CCTTATCCAGCTTTCCTTGGTC	
*POB3*	*Pyrenophora tritici-repentis* (XP_001937502.1)	653/417	V1908 (f): CACAGTGGAGCAAGGCATCC	HQ834399–HQ834414/HQ834415
			V1909 (r): ACATACCTAGGCGTCAAGTGC	
*SRP72*	*A. dermatitidis* (EEQ90678.1)	941/640	V1927 (f): AAGGGAAGGTTGGAGAGACTC	HQ834416–HQ834431/HQ834432
			V1895 (r): CAAGCAGCATTGTACGCCGTC	
*VPS13*	*Verticillium albo-atrum* (XP_003001174.1)	665/545	V1922 (f): GAGACAACGCTTGTTTGCAAGG	HQ834433–HQ834448/HQ834449
			V1923 (r): ACATGCGTCGTTCCAAGATCTG	

*Genes: *ALR*, α-L-rhamnosidase; *Bpntase*, 3′(2′),5′-bisphosphate nucleotidase; *DHC1*, Dynein heavy chain; *GPHN*, Gephyrin, molybdenum cofactor biosynthesis protein; *PCS*, peroxisomal-coenzyme A synthetase; *POB3*, FACT complex subunit; *SRP72*, signal recognition particle protein 72; *VPS13*, vacuolar protein sorting-associated protein. †f, forward; r, reverse.

To obtain DNA sequences from 1 *G. pannorum* and 16 *G. destructans* isolates, we prepared genomic DNA from mycelia grown in yeast extract peptone dextrose broth through conventional glass bead treatment and phenol-chloroform extraction and then ethanol precipitation (7). AccuTaq LA DNA Polymerase (Sigma-Aldrich, St. Louis, MO, USA) was used for PCR: 3 min initial denaturation at 94°C, 35 amplification cycles with a 15-sec denaturation at 94°C, 30-sec annealing at 55°C, and 1-min extension at 68°C and a 5-min final extension at 68°C. PCR products were treated with ExoSAP-IT (USB Corp., Cleveland, OH, USA) before sequencing. Both strands of amplicons were sequenced by the same primers used for PCR amplification (Table 2). A database was created by using Microsoft Access (Microsoft, Redmond, WA, USA) to deposit and analyze the sequences. Nucleotide sequences were aligned with ClustalW version 1.4 (www.clustal.org) and edited with MacVector 7.1.1 software (Accelrys, San Diego, CA, USA). Phylogenetic analyses were done by using PAUP 4.0 (8) and MEGA 4 (9).

**Figure 1:**
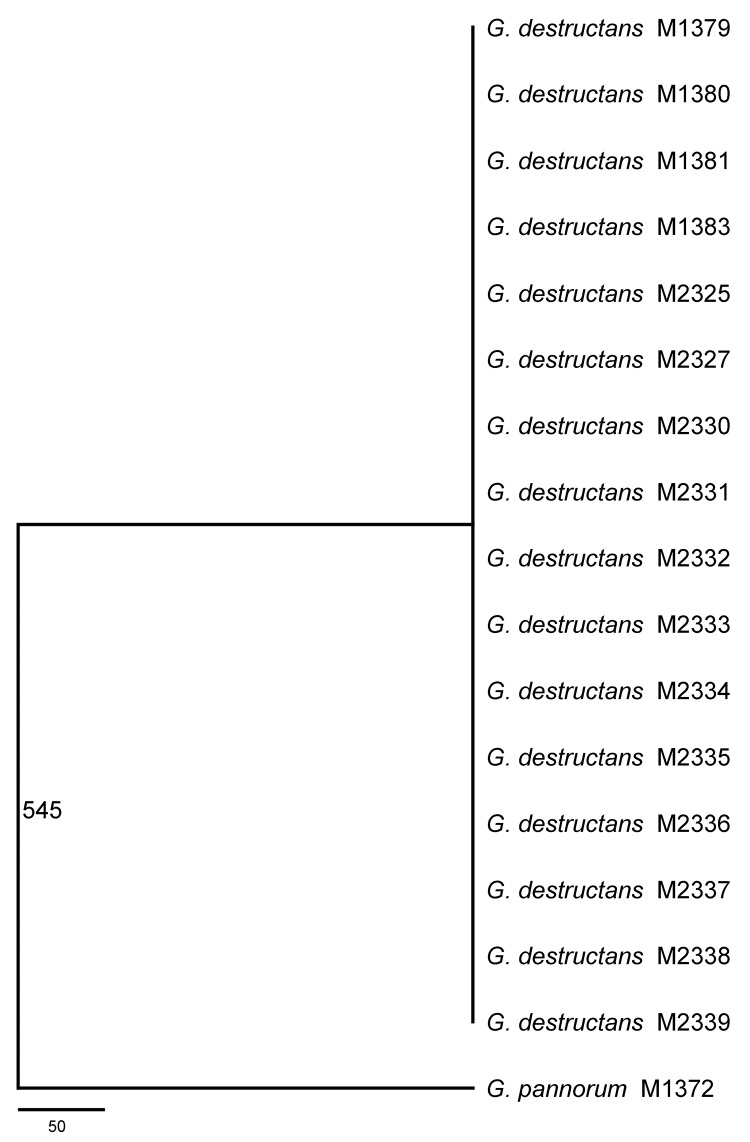
Consensus maximum-parsimony tree derived from analyzing 8 concatenated gene fragments including a total of 4,470 aligned nucleotides by using PAUP* 4.0 (*8*). The number 545 on the branch indicates the total number of variable nucleotide positions (out of the 4,470 nt) separating *Geomyces pannorum* M1372 from the clonal genotype of *G. destructans* identified here. Fifty of the 545 variable sites correspond to insertions and deletions. Scale bar indicates number of nucleotide substitutions per site.

We cloned and sequenced ≈200 Kb of the *G. destructans* genome and identified genes involved in a variety of cellular processes and metabolic pathways (Table 2). DNA sequence typing by using 8 gene fragments showed that all 16 *G. destructans* isolates had identical nucleotide sequences at all 8 sequenced gene fragments but were distinct from *G. pannorum* sequences. A maximum-parsimony tree generated from the 8 concatenated gene fragments indicated a single, clonal genotype for the 16 *G. destructans* strains (Figure 1). This consensus tree included 4,470 aligned nucleotides from all targeted gene sequences with 545 variable sites that separate the *G. destructans* clonal genotype from *G. pannorum*. Further analyses of the same concatenated gene fragments with exclusion of 50 insertions and deletions between *G. destructans* and *G. pannorum* yielded a tree with a shorter length (495 steps instead of 545 steps) but an identical topology (Technical Appendix Figure 1). This pattern remained unchanged when different phylogenetics models were used for analysis (Technical Appendix Figure 2). The lack of polymorphism among the 16 *G. destructans* isolates was unlikely because of evolutionary constraint at the sequenced gene fragments. We found many synonymous and nonsynonymous substitutions in target genes among a diversity of fungal species, including between *G. destructans* and *G. pannorum* (10) (Technical Appendix Figure 3).

**Figure 2:**
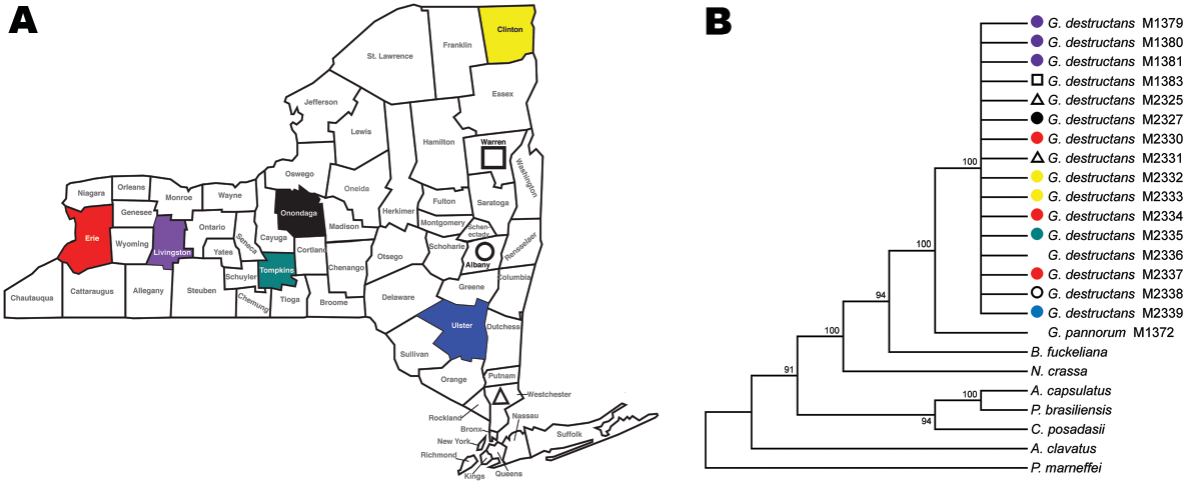
Collection sites in New York counties (A) are color-matched with respective *Geomyces destructans* isolates in maximum-parsimony tree based on nucleotide sequence of the VPS13 gene (B). The tree was constructed with MEGA4 (*9*) by using 450 nt and bootstrap test with 500 replicates. In addition to *G. destructans* and *G. pannorum*, fungi analyzed were *Ajellomyces capsulatus* (AAJI01000550.1), *Aspergillus clavatus* NRRL 1 (AAKD03000035.1), *Botryotinia fuckeliana* B05.10 (AAID01002173.1), *Coccidioides posadasii* C735 delta SOWgp (ACFW01000049.1), *Neurospora crassa* OR74A (AABX02000023.1), *Paracoccidioides brasiliensis* Pb01 (ABKH01000209.1), and *Penicillium marneffei* ATCC 18224 (ABAR01000009.1).

## Conclusions

Our finding of a single clonal genotype in *G. destructans* population fits well with the rapid spread of geomycosis in New York (Figure 2). Our sampling population covered both spatial and temporal dimensions, and the numbers of isolates analyzed were adequate in view of difficulties encountered in obtaining pure isolations of *G. destructans* (11). Although the affected New York sites are separated by sizable distances and include geographic barriers, a role for the natural dissemination of the fungus through air, soil, and water cannot be ruled out. Indeed, several fungi with geographic distributions similar to that in our study have shown major genetic variation among strains (12,13). It is also possible that humans and/or animals contributed to the rapid clonal dispersal. In such a scenario, the diseased or asymptomatic bats might act as carriers of the fungus by their migration into new hibernation sites where new animals get infected and the dissemination cycle continues (4). Similarly, the likely roles played by humans and/or other animals in the transfer of the fungal propagules from an affected site to a clean one cannot be ruled out from our data.

Virulent clones of human and plant pathogenic fungi that spread rapidly among affected populations have been recognized with increasing frequency in recent years (12,14). However, other pathogens, such as the frog-killing fungus *Batrachochytrium dendrobatidis*, have emerged with both clonal and recombining populations (13). Our data do not eliminate the possibility that the *G. destructans* population undergoes recombination in nature. This process to generate genetic variability would require some form of sexual reproduction, which remains unknown in *G. destructans.* In addition, the fungus might have both asexual and sexual modes in its saprobic life elsewhere in nature, but it exists only in asexual mode on bats (15).

In conclusion, our data suggest that a single clonal genotype of *G. destructans* has spread among affected bats in New York. This finding might be helpful for the professionals involved in devising control measures. Many outstanding questions remain about the origin of *G. destructans*, its migration, and reproduction, all of which will require concerted efforts if we are to save bats from predicted extinction (*3*).

## Supplementary Material

Technical Appendix Maximum-parsimony trees figures and Multiple alignments of 8 target gene fragments figure.
