# Supplementary material for: Clonal Genotype of Geomyces destructans among Bats with White Nose Syndrome, New York, USA
Source: Emerg Infect Dis. 2011 Jul;17(7):1273–6. doi: 10.3201/eid1707.102056 (PMC3381392; doi:10.3201/eid1707.102056)
Supplement: Technical Appendix — Maximum-parsimony trees figures and Multiple alignments of 8 target gene fragments figure. [file 10-2056-Techapp.pdf]

Article DOI: 10.3201/eid1707.102056

Clonal Genotype of *Geomyces destructans* among Bats with White Nose Syndrome, New York, USA

Technical Appendix

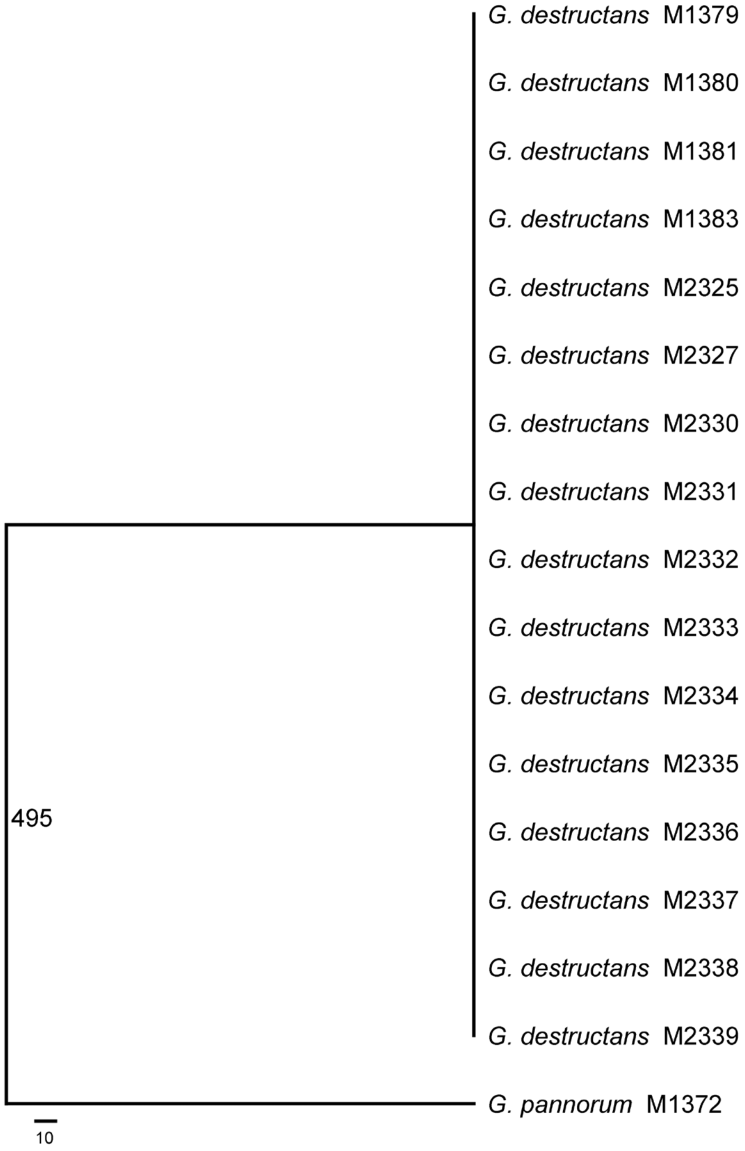

Technical Appendix Figure 1. Maximum-parsimony tree derived from analyzing 8 concatenated gene fragments. A total of 4,470 aligned nucleotides were analyzed by using PAUP* 4.0 (*8*). The number 495 on the branch indicates the number of different nucleotides (out of the 4,470 nt) separating *Geomyces pannorum* M1372 from the clonal genotype of *G. destructans* identified here. Insertions and deletions are excluded from this tree. Scale bar indicates number of nucleotide substitutions per site.

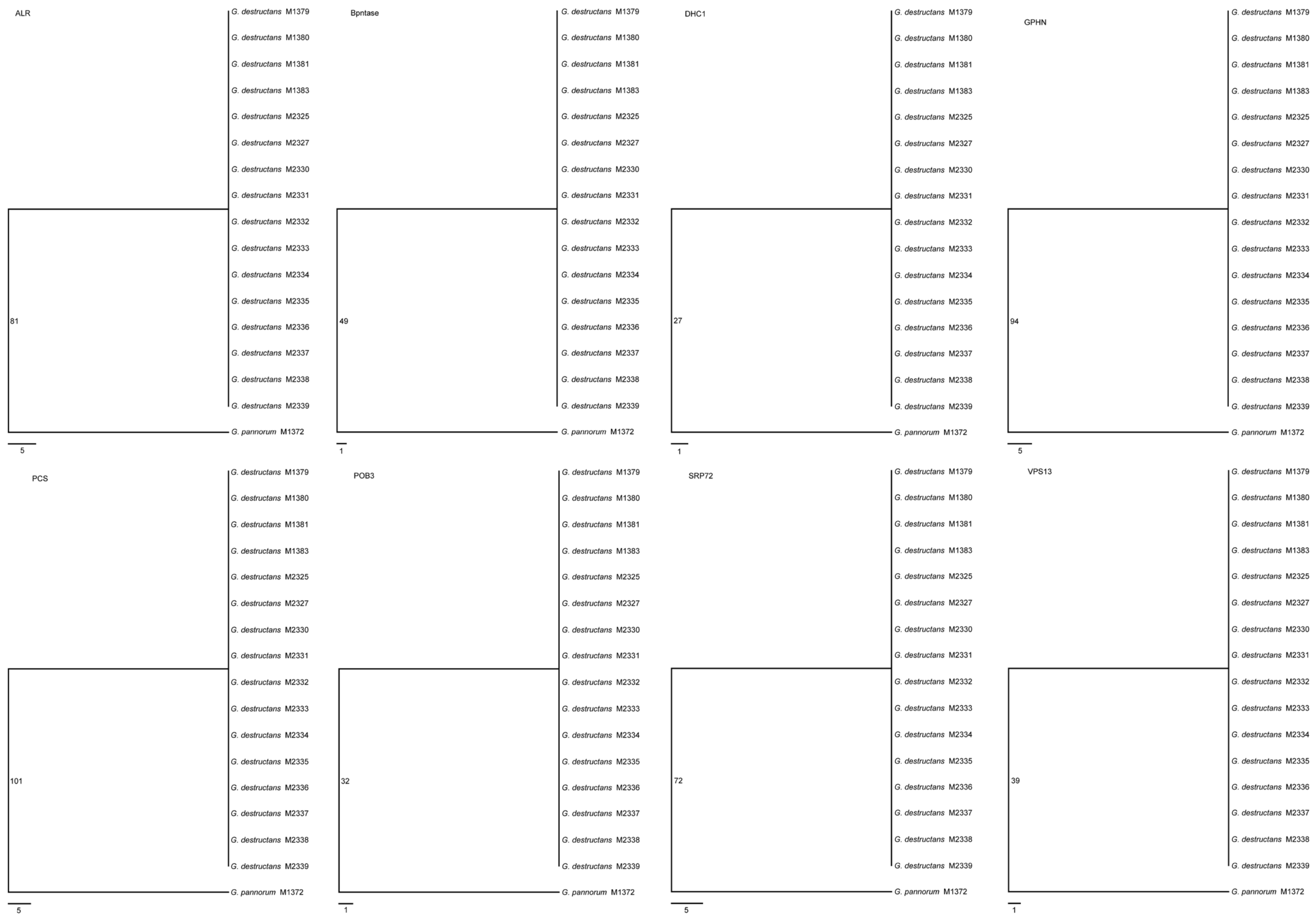

Technical Appendix Figure 2. Maximum parsimony trees derived from individual target sequences. Alpha-L-rhamnosidase (*ALR*), 530 aligned nucleotides with 81 nt difference between *Geomyces destructans* and *G. pannorum*; 3′(2′),5′-bisphosphate nucleotidase (Bpntase), 667 nt/49

nucleotide difference; dynein (*DHC1*), 417 nt /27 difference; gephyrin molybdenum cofactor biosynthesis protein (*GPHN*), 522 nt/94 nt difference; peroxisomal-coenzyme A synthetase (*PCS*), 719 nt/101 nt difference; FACT complex subunit (*POB3*), 413 nt/32 nt difference; signal recognition particle protein 72 (*SRP72*), 613 nt/72 nt difference; vacuolar protein sorting-associated protein (*VPS13*), 527 nt/39 nt difference. The tree topologies were identical when neighbor-joining methods were used for analysis. Scale bars indicate number of nucleotide substitutions per site.

Technical Appendix Figure 3 (below). Multiple alignments of 8 target gene fragments. Synonymous substitutions are shaded light gray while nonsynonymous substitution are dark gray and amino acid changes are highlighted in red. A smaller fragment of nucleotides used in phylogenetics construct was used in this comparison. Putative amino acid sequences for *Geomyces destructans* and *G. pannorum* were deduced by using EMBOSS Transeq (EMBL-EBI, Cambridgeshire, UK). Homologous sequences from either *Ajellomyces dermatiditis*, *Arthroderma otae*, *Aspergillus nidulans*, *Aspergillus clavatus*, *Botryotinia fuckeliana*, *Glomerella graminicola*, *Nectria hematococca*), *Sclerotinia sclerotiorum,* and *Verticillium albo-atrum* were included in this comparison.

ALR (Alpha-L-rhamnosidase): □450 bases aligned (Gd = *Geomyces destructans*, Gp = *Geomyces pannorum*
An = *Aspergillus nidulans*, Va = *Verticillium albo-atrum*)

```
Gd-DNA                        CTC TCT ACA AAA CTC TTG GCA CCG GAT GCG CCG CCC GTT ACG GTC
Gp-DNA                        CTC TCT ACA AAA CTC ATC GCT CCA GAT GCG CCA CCC GTC ACT GTG
An-DNA                        CCA GAG ACG CAG CTG GTG GCG CCA AAC GCG CCA CCA GTG CGA GTG
Va-DNA                        GAT ACC GTC CAG CTC CAT GCG GGT GAC GCC GAG CCT GTT CGA CGC
Gd-PRO                                     K   L   L   A   P   D   A   P   P   V   T   V
Gp-PRO                                     K   L   I   A   P   D   A   P   P   V   T   V
An-PRO                                     Q   L   V   A   P   N   A   P   P   V   R   V
Va-PRO                                     Q   L   H   A   G   D   A   E   P   V   R   R

Gd-DNA        ACA GAA GAG GTC AAC CCA GTC GAT ATT ATC AAG ACA AAG TCC GGG AAA ACA GTC ATT GAT
Gp-DNA        ACA GAA GAG CTG AGC CCA ACT GAG ATT ATC AAG ACA AAG TCC GGC AAA ACG GTC ATT GAC
An-DNA        ACC GAA GTG GTC AAT CCA GTT GAA ATC ATT CGC ACG CCG TCC GGC AAA GTA ATC ATT GAT
Va-DNA        CTC GAA ATC GTC AAG CCC ATC GAA AAG ATC ACG ACC CCC TCC GGA AAG ACG GTG CTT GAC
Gd-PRO         T   E   E   V   N   P   V   D   I   I   K   T   K   S   G   K   T   V   I   D
Gp-PRO         T   E   E   L   S   P   T   E   I   I   K   T   K   S   G   K   T   V   I   D
```

```
An-PRO     T   E   V   V   N   P   V   E   I   I   R   T   P   S   G   K   V   I   I   D
Va-PRO     L   E   I   V   K   P   I   E   K   I   T   T   P   S   G   K   T   V   L   D

Gd-DNA    TTT GGG CAG AAC CTG GTA GGC AAG CTT CGT GTC AGC TCC GTC CGA CTC CCC GCG GGT CA
Gp-DNA    TTT GGG CAG AAC CTC GTA GGG AAA CTT CGT GTC AAC TCC GTC CGA CTC CCT GCC GGT GA
An-DNA    TTT GGG CAA AAC CTC GTT GGC CGT GTC CGG ATC CGC TCC GTA AAG AAG ACC GTG GGC CA
Va-DNA    TTT GGG CAG AAC CTC GTA GGC TAC CTT CGG GTC AAC AAA GTC AGG GGT CCC CGC GGC CA
Gd-PRO     F   G   Q   N   L   V   G   K   L   R   V   S   S   V   R   L   P   A   G   Q
Gp-PRO     F   G   Q   N   L   V   G   K   L   R   V   N   S   V   R   L   P   A   G   E
An-PRO     F   G   Q   N   L   V   G   R   V   R   I   R   S   V   K   K   T   V   G   H
Va-PRO     F   G   Q   N   L   V   G   Y   L   R   V   N   K   V   R   G   P   R   G   H

Gd-DNA    G AAG ATC TCA TTT ACA CAT GTC GAA GTG CTC GAG AAT GGC GAA ATC GGC ACA CGT CCG CT
Gp-DNA    G AAA ATC ACA TTT ACA CAC GTC GAG GTA CTC GAG AAT GGC GAA ATT AGC ACC CGA CCG CT
An-DNA    C TCT ATC ATT CTC AAG CAT GCG GAA GTG CTC GAG AAT AGC GAA CTT GGA ACG CGT CCG CT
Va-DNA    T AAA ATC ACC CTG CTG CAC GCC GAG GTT CTC GAG AAG GGC GAG CTT GGC ATC CGG CCG CT
Gd-PRO       K   I   S   F   T   H   V   E   V   L   E   N   G   E   I   G   T   R   P   L
Gp-PRO       K   I   T   F   T   H   V   E   V   L   E   N   G   E   I   S   T   R   P   L
An-PRO       S   I   I   L   K   H   A   E   V   L   E   N   S   E   L   G   T   R   P   L
Va-PRO       K   I   T   L   L   H   A   E   V   L   E   K   G   E   L   G   I   R   P   L

Gd-DNA    T CGA GGA GCA GTC TGC GTT GAT ACT ATT GTC TTT TCT GAA AAG GAG CTC CGC GGC -=
Gp-DNA    T CGA GAA GCA GTA CCC GTC GAT ACT GTC ATC TTT TCT GAT AAC GAG CTC CTG AAC --
An-DNA    G CGA GTG GCC AAG GCC CAG GAC GAG ATC ATC TCA GCA GGA CAG GAG ATT TGT GAC --
Va-DNA    G CGT GAC TGC AAG GCC CGG GAC ATC TAC ACC CTC TGT GGT GAT GAG GCT GGC GAA TC
Gd-PRO       R   G   A   V   C   V   D   T   I   V   F   S   E   K   E   L   R   G   -
Gp-PRO       R   E   A   V   P   V   D   T   V   I   F   S   D   N   E   L   L   N   -
An-PRO       R   V   A   K   A   Q   D   E   I   I   S   A   G   Q   E   I   C   D   -
Va-PRO       R   D   C   K   A   R   D   I   Y   T   L   C   G   D   E   A   G   E   S

Gd-DNA    - TGG TCG CCG AAA TTC ACA TTC CAC GGC TTC CAG TAC GTG CAG GTT GAA GGG TGG CCA GC
Gp-DNA    - TGG TCC CCA AAA TTC ACA TTT CAT GGT TTC CAG TAC GTG CAA GTT GAT GGG TGG CCA GC
An-DNA    - TGG GCT CCT AGC TTT ACT TTC CAT GGC TTC CGG TAT GTT CAG GTT GAT GGA TGG AGC --
Va-DNA    G TAC GAG CCT CGC TTC ACC TTC CAC GGC TTT CGC TAT GCC CAG GTC GAC GAC TGG CCA --
Gd-PRO       W   S   P   K   F   T   F   H   G   F   Q   Y   V   Q   V   E   G   W   P   A
Gp-PRO       W   S   P   K   F   T   F   H   G   F   Q   Y   V   Q   V   D   G   W   P   A
An-PRO       W   A   P   S   F   T   F   H   G   F   R   Y   V   Q   V   D   G   W   S   -
Va-PRO       Y   E   P   R   F   T   F   H   G   F   R   Y   A   Q   V   D   D   W   P   -
```

```
Gd-DNA          A ACT GCG GAT GCT GAA CTC CCT --- TAT AAG TCT GAC TTT ACT GCG CTA GTC ATG CAT 445
Gp-DNA          A ACT GCG GAT GCT GAA CTC CCA --- TCT TTG TCT GAC TTC ACT GCA CTA GTC ATG CAT 452
An-DNA          - CCT GAA GAC GCC GAC ACC CCT CTT ACT CTA CAA AGC CTG ACC GCC GAA GTC ATG CAT 444
Va-DNA          - TCC GCA GAT ATT GAC --- --- --- ATC CTG GAC TCA CTC GAG GCT GTG GTC TGC AAT 459
Gd-PRO             T   A   D   A   E   L   P   -   Y   K   S   D   F   T   A   L   V   M   H
Gp-PRO             T   A   D   A   E   L   P   -   S   L   S   D   F   T   A   L   V   M   H
An-PRO             P   E   D   A   D   T   P   L   T   L   Q   S   L   T   A   E   V   M   H
Va-PRO             S   A   D   I   D   -   -   -   I   L   D   S   L   E   A   V   V   C   N
```

BPntase (3'(2'),5'-bisphosphate nucleotidase): ≈750 bases aligned; Alignment shows 3' region of the protein and the 3' UTR (Ss = *Sclerotinia sclerotiorum*; Af = *Aspergillus fumigatus*)

```
Gd-DNA                                                  AG AGC CAG CCG ATC CGC ATG AGC GAG AAG A
Gp-DNA                                                  AG AGC CAG CCA ATC CGC ATG AGC GAG AAG A
Ss-DNA                                                  AG GGT CAA TCT ATT CAA ATG AAG CCA GTT A
Af-DNA                                                  AG AGC AAG CCC ATC TCG ATG CGT CCC GTT C
Gd-PRO                                                      S   Q   P   I   R   M   S   E   K
Gp-PRO                                                      S   Q   P   I   R   M   S   E   K
Ss-PRO                                                      G   Q   S   I   Q   M   K   P   V
Af-PRO                                                      S   K   P   I   S   M   R   P   V
```

```
Gd-DNA          AG GAC ATC ACC GAT GCC ACT TTC TGC GAG AGT GTT GAG GCT GGC CAC TCA TCT CAC GAC G
Gp-DNA          AG GAT ATC ACC GAT GCC ACT TTC TGC GAG AGT GTT GAG GCT GGC CAC TCG TCG CAT GAC G
Ss-DNA          CG GAT TTA AGT CAA GCT ACA TTC TGT GAG AGT GTT GAG GCA GGT CAC TCT TCC CAT GGC G
Af-DNA          CG GAT ATA AAA CAG GCT GTC TTC TGT GAA GGA GTT GAG GCT GCC CAC TCT GCT CAA GGC G
Gd-PRO          K   D   I   T   D   A   T   F   C   E   S   V   E   A   G   H   S   S   H   D
Gp-PRO          K   D   I   T   D   A   T   F   C   E   S   V   E   A   G   H   S   S   H   D
Ss-PRO          T   D   L   S   Q   A   T   F   C   E   S   V   E   A   G   H   S   S   H   G
Af-PRO          P   D   I   K   Q   A   V   F   C   E   G   V   E   A   A   H   S   A   Q   G
```

```
Gd-DNA          AC CAA TCT CAG ATC GCC CAG AAG CTG CAG ATC AGC AAG CCG AGC GTG CGG ATG GAT TCG C
Gp-DNA          AC CAG TCT CAG ATC GCA CAG AAG CTG CAG ATT AGC AAG CCA AGC GTG CGA ATG GAT TCG C
Ss-DNA          AT CAA CAT GCC ATT GCT ACC AAA TTG GGT GTT ACC AAG GCT AGT GTC CGA ATG GAT TCA C
Af-DNA          AC AAC GCT GCC GTC GCT CAG CTC CTG GGT ATC ACC TCC CCC AGC GTG CGA CTC GAC TCG C
Gd-PRO          D   Q   S   Q   I   A   Q   K   L   Q   I   S   K   P   S   V   R   M   D   S
Gp-PRO          D   Q   S   Q   I   A   Q   K   L   Q   I   S   K   P   S   V   R   M   D   S
```

```
Ss-PRO    D  Q  H  A  I  A  T  K  L  G  V  T  K  A  S  V  R  M  D  S
Af-PRO    D  N  A  A  V  A  Q  L  L  G  I  T  S  P  S  V  R  L  D  S

Gd-DNA    AG GCC AAG TAT GCC TCG ATC GCC CGT GGC GCT GGG GAT ATT TAC CTG AGA CTT CCA ACC A
Gp-DNA    AG GCA AAG TAT GCC TCG ATC GCT CGT GGT GCG GGG GAT ATT TAC CTC AGA CTT CCA ACC A
Ss-DNA    AA GCT AAA TAT GGA TCG ATC GCG AGA GGT GCT GGA GAC ATT TAC CTT AGA CTT CCC GTT A
Af-DNA    AG GCC AAG TAC TGC TCA ATT GCT AGA GGC GCA GGC GAT ATC TAC CTA CGG CTA CCT GTC A
Gd-PRO    Q  A  K  Y  A  S  I  A  R  G  A  G  D  I  Y  L  R  L  P  T
Gp-PRO    Q  A  K  Y  A  S  I  A  R  G  A  G  D  I  Y  L  R  L  P  T
Ss-PRO    Q  A  K  Y  G  S  I  A  R  G  A  G  D  I  Y  L  R  L  P  V
Af-PRO    Q  A  K  Y  C  S  I  A  R  G  A  G  D  I  Y  L  R  L  P  V

Gd-DNA    GC GCA ACG TAC CAA GAG AAG ATC TGG GAT CAC GCT GCT GGA GAC CTG ATT GTT CGA GAG G
Gp-DNA    GC GCA ACG TAC CAG GAG AAG ATC TGG GAT CAC GCT GCT GGA GAC CTG ATT GTT AGG GAG G
Ss-DNA    GC GCG ACT TAC CAA GAG AAG ATT TGG GAT CAT GCT GCT GGA GAT CTT ATT GTA AGA GAG G
Af-DNA    GA AAG GAC TAC CAG GAG AAG ATC TGG GAT CAT GCG GCT GGC GAT CTC ATT GTC CGC GAG G
Gd-PRO    S  A  T  Y  Q  E  K  I  W  D  H  A  A  G  D  L  I  V  R  E
Gp-PRO    S  A  T  Y  Q  E  K  I  W  D  H  A  A  G  D  L  I  V  R  E
Ss-PRO    S  A  T  Y  Q  E  K  I  W  D  H  A  A  G  D  L  I  V  R  E
Af-PRO    R  K  D  Y  Q  E  K  I  W  D  H  A  A  G  D  L  I  V  R  E

Gd-DNA    CG GGA GGA CAG GTT ACG GAT TCA CTA GGC AGA CGT CTG GAT TTC AGC AAG GGT AGA ACT T
Gp-DNA    CA GGA GGA CAG GTT ACG GAT TCA CTG GGC AGA CGT CTG GAT TTC AGC AAG GGT AGA ACC T
Ss-DNA    CA GGT GGA CAG GTT ACT GAT TCT CTC GGA CGA AGA TTG GAT TTC AGC AAG GGA AGA ACT T
Af-DNA    CT GGT GGA CAA GTG ACC GAT ATC TAT GGC CAG CGC TTG GAT TTC AGC AAG GGA CGC ACT T
Gd-PRO    A  G  G  Q  V  T  D  S  L  G  R  R  L  D  F  S  K  G  R  T
Gp-PRO    A  G  G  Q  V  T  D  S  L  G  R  R  L  D  F  S  K  G  R  T
Ss-PRO    A  G  G  Q  V  T  D  S  L  G  R  R  L  D  F  S  K  G  R  T
Af-PRO    A  G  G  Q  V  T  D  I  Y  G  Q  R  L  D  F  S  K  G  R  T

Gd-DNA    TG GCT GAG AAT AAG GGT GTC GTT GCC GCA CCA CAG GCT CTA CAC GCA CGA GTC CTT GAG G
Gp-DNA    TG GCT GAG AAT AAG GGT GTC GTT GCC GCA CCA CAG GCT CTA CAT GCA CGA GTC CTT GAG G
Ss-DNA    TG GCT GAA AAC AAG GGT GTC GTT GCA GCT CCC GCA GCC ATT CAC GAC CAC GTT TTG GAG G
Af-DNA    TG GCT GCC AAC AAG GGA GTC GTC GCC GCT CCA GAG GCC ATC CAG GAC CAG GTT ATT AGT G
Gd-PRO    L  A  E  N  K  G  V  V  A  A  P  Q  A  L  H  A  R  V  L  E
Gp-PRO    L  A  E  N  K  G  V  V  A  A  P  Q  A  L  H  A  R  V  L  E
Ss-PRO    L  A  E  N  K  G  V  V  A  A  P  A  A  I  H  D  H  V  L  E
Af-PRO    L  A  A  N  K  G  V  V  A  A  P  E  A  I  Q  D  Q  V  I  S
```

```
Gd-DNA          TT GTC AAG GAG GTC CTT GGC AAG AAG GGA AAC CTA TAG --------CAGATATGA
Gp-DNA          TT GTC AAA GAA GTC CTG GGC AAG AAA GGA AAC CTA TAG --------TAGATATGA
Ss-DNA          TG GTT AAG GAG GTT TTG GGG CCC AAG AAA TAA ---ATTGTTCATATATTCGTCACAA
Af-DNA          CC GTC AAG ACG GTC CTG AAG CTA TGA AGGATGACGCGAACGGACCAACTGTTGTATCGA
Gd-PRO          V   V   K   E   V   L   G   K   K   G   N   L   *
Gp-PRO          V   V   K   E   V   L   G   K   K   G   N   L   *
Ss-PRO          V   V   K   E   V   L   G   P   K   K   *
Af-PRO          A   V   K   T   V   L   K   L   *

Gd-DNA          ACAATTGAAATAGATTCTA---CATATCGTATAACT-ATCCGCTTGG-GACCCTCCAATT
Gp-DNA          ACAGCTG---TAGATTCTA---CATATCGTATAACT-ATCCGCTTGA-GACTCCACAATT
Ss-DNA          ATAAAAGCCATGATACCTTGATCCACTTGTACATCG-ACACCATAGATATATCTACAATA
Af-DNA          ATCACTAAACCCGTTCTAA--ACCTTTGTTAGATCCTGCACGCTTGATGCGCCTAAAAGT

Gd-DNA          CTATGACA-------ATATAAG---AGCAATATAATAC-ATGGACG--AATCGTCTATTT
Gp-DNA          CTATGACA-------ATATAAG---AACAATATAATAC-ATGGACG--AATCGTCTATTT
Ss-DNA          CCTACAAATG-----ACATTAT---CTCTAGAGATTAT-TTGGATATTCATCCACTACTT
Af-DNA          ACATGAGATAGATACCAATTGGGAAAACAGGAGACTCTGCTGGAAT--ATACATGTACAG

Gd-DNA          GATCTTCTTGGCCTTCAACCTGATCGTCTCACCGTTGACGAAAATTCCCTTCCCCT--TG
Gp-DNA          GATCTTCTTCGCCTTCAGCTTGATCGTCTCGCCATTGACGAAGATTCCCTTCCCCT--TA
Ss-DNA          AATCTTCTTATTCTTCAACTTAATCTCCTCTCCATTGACAAAGATTCCCTTCCCCT--TA
Af-DNA          AAACAGCCCCAGACCCTGTTCCAAGAAAATGCCAAAGTAAAATAGAAACCACCCCAGATC

Gd-DNA          TATGGCTCAGGCTTCCTCCACTGGCGGATTTCCGCAGCAAACTGCAGAACCACTT---CC
Gp-DNA          TATGGCTCAGGCTTCCTCCATTCACGAATTTCCGCAGCGAACTGGAGAACAACCT---CC
Ss-DNA          TACGGTTCTGGAACTCTCCACTTTCTAATTTGCGCAGCGAAAAGTAATACAACTT---CC
Af-DNA          ACCGAATCTT-CTTGGCCTTCAGTCTGATTGTCTCTCCGTTCACAAAAATACCCTTGCCC

Gd-DNA          TTCTCCGGGCCCT-CCAGCAATATTCTCGTTGGCTGCGGTGTGCTGGCCGTCATGCCCTT
Gp-DNA          TTCTCTGGGCCCT-CCAGCAATATTCTCGTTGGCTGCGGTGTGCTAGCCGTCATGCCCTT
Ss-DNA          TTCTCTGGTCCTT-CTAACAATATACGTGTAGGTTGTGGTGTGCTCGCTTTCACAAATCT
Af-DNA          TTGTAAGGCTCTGGCTTCCGCCATTCTC-TGATTTCCGCCGCGAATTGTGTCACAACGTT

Gd-DNA          CGGCACTGGCAACTCGA-TAGGATGTGAGTAG 744
Gp-DNA          CGGCACTGGCAACTCGA-TTGGATGTGAGTAC 741
```

```
Ss-DNA          TGGAACGGGTAATTCGA-TAGGATGAGAGTAC 759
Af-DNA          TTTGTCGACTCCCTCAAGCAGAATACGAGTAG 775
```

DHC1 (Cytoplasmic dynein heavy chain): 418 bases aligned (Ss = *Sclerotinia sclerotiorum*, Bf = *Botryotinia fuckeliana*)

```
Ss-DNA          GAT GCT GAA TAC CTA GAT CCA ATC CTT AAC CAC GTT CTT AAC AAA GAG TAT CAA AAG ACT 60
Bf-DNA          GAT GCC GAA TAC CTG GAC CCA ATT CTT AAC CAC GTT CTC AAC AAA GAG TAC CAA AAG ACT 60
Gd-DNA          GAT GCC GAG CAT TTG GAT CCA ATC CTT AAC CAC GTC CTC AAC AAG GAA TAC CAA AAG ACT 60
Gp-DNA          GAT GCC GAG CAT TTG GAT CCG ATC CTT AAT CAT GTC CTC AAC AAG GAG TAC CAA AAG ACT 60
Ss-PRO           D   A   E   Y   L   D   P   I   L   N   H   V   L   N   K   E   Y   Q   K   T
Bf-PRO           D   A   E   Y   L   D   P   I   L   N   H   V   L   N   K   E   Y   Q   K   T
Gd-PRO           D   A   E   H   L   D   P   I   L   N   H   V   L   N   K   E   Y   Q   K   T
Gp-PRO           D   A   E   H   L   D   P   I   L   N   H   V   L   N   K   E   Y   Q   K   T
```

```
Ss-DNA          GGT GGA CGT GTT CTT ATT CAG CTT GGT AAG CAA GAA ATT GAC TTT TCG CCC GCA TTT AAG 120
Bf-DNA          GGT GGG CGT GTT CTC ATC CAG CTT GGA AAG CAA GAA ATC GAT TTC TCG CCC GCA TTC AAG 120
Gd-DNA          GGT GGA CGT GTC CTC ATC CAG CTC GGT AAA CAA GAG ATC GAT TTC TCC CCA GCC TTC AAG 120
Gp-DNA          GGC GGG CGT GTT CTC ATC CAG CTT GGC AAA CAA GAA ATC GAT TTC TCC CCA GCC TTC AAG 120
Ss-PRO           G   G   R   V   L   I   Q   L   G   K   Q   E   I   D   F   S   P   A   F   K
Bf-PRO           G   G   R   V   L   I   Q   L   G   K   Q   E   I   D   F   S   P   A   F   K
Gd-PRO           G   G   R   V   L   I   Q   L   G   K   Q   E   I   D   F   S   P   A   F   K
Gp-PRO           G   G   R   V   L   I   Q   L   G   K   Q   E   I   D   F   S   P   A   F   K
```

```
Ss-DNA          ATC TAC CTT TCC ACT AGA GAT CCA TCT GCA ACA TTC GCA CCG GAT ATT TGC AGT CGG ACA 180
Bf-DNA          ATT TAC CTT TCC ACC AGA GAT CCA TCT GCA ACA TTC GCA CCG GAC ATT TGC AGT CGC ACA 180
Gd-DNA          ATC TAC CTC TCG ACC AGA GAT CCA TCT GCT ACG TTT GCA CCA GAC ATA TGC AGT CGC ACA 180
Gp-DNA          ATC TAT CTC TCA ACC AGA GAT CCA TCT GCT ACA TTT GCA CCA GAT GTA TGC AGT CGC ACA 180
Ss-PRO           I   Y   L   S   T   R   D   P   S   A   T   F   A   P   D   I   C   S   R   T
Bf-PRO           I   Y   L   S   T   R   D   P   S   A   T   F   A   P   D   I   C   S   R   T
Gd-PRO           I   Y   L   S   T   R   D   P   S   A   T   F   A   P   D   I   C   S   R   T
Gp-PRO           I   Y   L   S   T   R   D   P   S   A   T   F   A   P   D   V   C   S   R   T
```

```
Ss-DNA          ACG TTT GTC AAT TTC ACT GTC ACA CAA AGC AGT TTA CAA ACA CAG TCA CTT AAT GAC GTC 240
Bf-DNA          ACA TTT GTT AAT TTC ACA GTC ACA CAG AGT AGT TTG CAA ACA CAG TCA CTC AAT GAC GTT 240
```

```
Gd-DNA    ACT TTC GTC AAT TTC ACC GTC ACC CAG AGC AGT CTC CAA ACG CAA TCA TTG AAC GAA GTC 240
Gp-DNA    ACC TTC GTG AAT TTC ACT GTC ACC CAG AGC AGT CTC CAA ACA CAA TCA TTG AAC GAA GTC 240
Ss-PRO     T   F   V   N   F   T   V   T   Q   S   S   L   Q   T   Q   S   L   N   D   V
Bf-PRO     T   F   V   N   F   T   V   T   Q   S   S   L   Q   T   Q   S   L   N   D   V
Gd-PRO     T   F   V   N   F   T   V   T   Q   S   S   L   Q   T   Q   S   L   N   E   V
Gp-PRO     T   F   V   N   F   T   V   T   Q   S   S   L   Q   T   Q   S   L   N   E   V

Ss-DNA    CTC AAA TCT GAG CGA CCT GAT GTG GAT GAG AGG CGC TCA AAT CTC ATC AAA TTA CAG GGT 300
Bf-DNA    CTC AAA TCC GAA CGA CCT GAT GTG GAC GAG AGA CGC TCT AAT CTC ATC AAG TTA CAA GGC 300
Gd-DNA    CTG AAA TCT GAG CGA CCT GAC GTG GAT GAG CGA AGA TCC AAC CTG ATC AAA TTA CAG GGA 300
Gp-DNA    CTG AAA TCT GAG CGA CCT GAC GTG GAT GAA CGA AGA TCC AAC CTG ATC AAA TTG CAG GGA 300
Ss-PRO     L   K   S   E   R   P   D   V   D   E   R   R   S   N   L   I   K   L   Q   G
Bf-PRO     L   K   S   E   R   P   D   V   D   E   R   R   S   N   L   I   K   L   Q   G
Gd-PRO     L   K   S   E   R   P   D   V   D   E   R   R   S   N   L   I   K   L   Q   G
Gp-PRO     L   K   S   E   R   P   D   V   D   E   R   R   S   N   L   I   K   L   Q   G

Ss-DNA    GAA TTC AAA GTT CAT CTT AGA CAG CTC GAG AAA CGC TTG TTG CAA GCA TTG AAC GAA TCA 360
Bf-DNA    GAA TTC AAA GTT CAC CTC AGG CAG CTC GAG AAG CGC TTG CTG CAA GCC CTG AAC GAA TCA 360
Gd-DNA    GAA TTC AAG GTC CAT CTT CGC CAA TTG GAG AAG CGT CTT TTG CAA GCT TTG AAC GAG TCC 360
Gp-DNA    GAA TTC AAG GTC CAC CTT CGC CAA TTG GAG AAG CGT CTT TTG CAG GCT TTG AAC GAG TCC 360
Ss-PRO     E   F   K   V   H   L   R   Q   L   E   K   R   L   L   Q   A   L   N   E   S
Bf-PRO     E   F   K   V   H   L   R   Q   L   E   K   R   L   L   Q   A   L   N   E   S
Gd-PRO     E   F   K   V   H   L   R   Q   L   E   K   R   L   L   Q   A   L   N   E   S
Gp-PRO     E   F   K   V   H   L   R   Q   L   E   K   R   L   L   Q   A   L   N   E   S

Ss-DNA    CGT GGC AAT ATT CTC GAT GAT GAT AAC GTC ATT GAA ACT CTC GAA ACT TTG AAG AAG G 418
Bf-DNA    CGT GGC AAT ATT CTT GAT GAT GAC AAC GTC ATT GAA ACT CTT GAG ACT TTG AAG AAG G 418
Gd-DNA    CGT GGC AAT ATC TTG GAT GAT GAC AAC GTC ATT GAG ACT CTC GAG ACG TTG AAG AAG G 418
Gp-DNA    CGT GGT AAT ATC TTG GAT GAT GAC AAC GTT ATT GAA ACT CTC GAA ACG TTG AAG AAG G 418
Ss-PRO     R   G   N   I   L   D   D   D   N   V   I   E   T   L   E   T   L   K   K
Bf-PRO     R   G   N   I   L   D   D   D   N   V   I   E   T   L   E   T   L   K   K
Gd-PRO     R   G   N   I   L   D   D   D   N   V   I   E   T   L   E   T   L   K   K
Gp-PRO     R   G   N   I   L   D   D   D   N   V   I   E   T   L   E   T   L   K   K
```

GPHN (Gephyrin): ~300 bases aligned; the alignment starts with non-coding miscellaneous feature

(Nh = *Nectria haematococca*, Ac = *Aspergillus clavatus*)

```
Gd-DNA      ATT----TC-CATTCTAACACGTAA-----------T ACA GTT GCA CTC ATG TCA CGC CC
Gp-DNA      ACT----TT-CATTCTAATTCGCAG-----------T ACA GTT GCA CTC ATG TCA CGT CC
Nh-DNA      ATC----TCATATTCTAATTCGAAACTGAT-CAAGCT TTA GTT GCA ATG ATG TCA CGC CC
Ac-DNA      ATTGCCCTTGAATCCAGAGCCATGACTGACATCTACT GTA GTT GCA ATG ATG TCT CGC CC
Gd-PRO                                                     A   L   M   S   R   P
Gp-PRO                                                     A   L   M   S   R   P
Nh-PRO                                                     A   M   M   S   R   P
Ac-PRO                                                     A   M   M   S   R   P

Gd-DNA      T GTT GCT GGT GTC CGC AAC AAG AGC ATC ATT ATC ACC CTC CCA GGC TCG CCA AAG GGC GC
Gp-DNA      T GTT GCT GGC GTC AGA AAC AAG AGT ATC ATT ATC ACT CTT CCA GGC TCG CCG AAG GGT GC
Nh-DNA      A GTG GCT GGC GTC CGT AAC CAG ACC ATC ATT GTC ACC TTA CCT GGC TCT CCC AAG GGT GC
Ac-DNA      C GTG GCG GGT GTC AGA GAT AAG TCA CTC CTG ATT ACA CTA CCA GGC TCG CCA AAG GGA GC
Gd-PRO         V   A   G   V   R   N   K   S   I   I   I   T   L   P   G   S   P   K   G   A
Gp-PRO         V   A   G   V   R   N   K   S   I   I   I   T   L   P   G   S   P   K   G   A
Nh-PRO         V   A   G   V   R   N   Q   T   I   I   V   T   L   P   G   S   P   K   G   A
Ac-PRO         V   A   G   V   R   D   K   S   L   L   I   T   L   P   G   S   P   K   G   A

Gd-DNA      C AAG GAG AAT CTC CAG TGC ATA TTA AAG CTC CTA CCT CAT GCA TGC TTG CAA GCA GCA G
Gp-DNA      G AAG GAG AAT CTC CAG TCA GTG CTG AAA CTT CTA CCA CAT GCT TGT TTG CAG GCA GCA G
Nh-DNA      C AGG GAG AAT CTC CAG GCC ATC GTC AAG ACT CTT CCT CAT GCT TGC CTC CAG GCG TCA G
Ac-DNA      C AAG GAA AAT CTT GAG GCA ATC ATC AAG CTT CTA CCG CAC GCT TGT ACT CAG GCG GCT G
Gd-PRO         K   E   N   L   Q   C   I   L   K   L   L   P   H   A   C   L   Q   A   A
Gp-PRO         K   E   N   L   Q   S   V   L   K   L   L   P   H   A   C   L   Q   A   A
Nh-PRO         R   E   N   L   Q   A   I   V   K   T   L   P   H   A   C   L   Q   A   S
Ac-PRO         K   E   N   L   E   A   I   I   K   L   L   P   H   A   C   T   Q   A   A

Gd-DNA      GG GCG GAC TCC AGA TCT CTA CAT GCT GGA GGC ATC AAA AAG CTG GAA TCG GAG GCT GGA A
Gp-DNA      GT GCC GAT TCG AGA TCT CTG CAT GCT GGT GGC ATC AAA AAG CTA GAA TCG GAG GCT GGG A
Nh-DNA      GG GCC AAC TCG AGA TCT CTC CAT GCT GGG GGA GTT AAG AAA CTA GAG TCA GAT GCA GGT T
Ac-DNA      GA GCA GAC TCG CGA GCT CTT CAT GCT GGC GGT GTC AAG AAG CTG GAA GCC GAA GCT GGT G
Gd-PRO      G   A   D   S   R   S   L   H   A   G   G   I   K   K   L   E   S   E   A   G
Gp-PRO      G   A   D   S   R   S   L   H   A   G   G   I   K   K   L   E   S   E   A   G
Nh-PRO      G   A   N   S   R   S   L   H   A   G   G   V   K   K   L   E   S   D   A   G
Ac-PRO      G   A   D   S   R   A   L   H   A   G   G   V   K   K   L   E   A   E   A   G
```

```
Gd-DNA         TA AGC TCT AGC GGA TCT CAC GCT GGC CAT CAC GGC TAT GGT CAC GGT CAC GAC CA 303
Gp-DNA         TA GGC TCT GGT GGA GCT CAT GCT GGA CAT CAT GGC CAC AGC CAT GGT CAC GGC CA 318
Nh-DNA         TA TCA GGT GGA AAG CCC CAA CCT GCT GCC AGC CAC AGT CAC GGA TGC CAC AGA CA 324
Ac-DNA         TA GCC CCC AAT GAT CCG CGC TCG GAA AGT CAA GGA CAG CAG CAT GAT CAC CAT CA 354
Gd-PRO         I   S   S   S   G   S   H   A   G   H   H   G   Y   G   H   G   H   D
Gp-PRO         I   G   S   G   G   A   H   A   G   H   H   G   H   S   H   G   H   G
Nh-PRO         L   S   G   G   K   P   Q   P   A   A   S   H   S   H   G   C   H   H
Ac-PRO         V   A   P   N   D   P   R   S   E   S   Q   G   Q   Q   H   D   H   H
```

PCS (Peroxisomal-coenzyme A synthetase): ~480 bases aligned; Alignment shows the exonic region and a predicted intronic region (Ac = *Aspergillus clavatus*; Ao = *Arthroderma otae*)

```
Gd-DNA                                        GTA CCC TTG ACA CAC A
Gp-DNA                                        GTG CCC TTG ACA CAC A
Ac-DNA                                        GTT CCC CTG ACG CAC A
Ao-DNA                                        GTA CCG CTC TCA CAT A
Gd-PRO                                         V   P   L   T   H
Gp-PRO                                         V   P   L   T   H
Ac-PRO                                         V   P   L   T   H
Ao-PRO                                         V   P   L   S   H

Gd-DNA         GG AAT TTG ACG AGG ACA ATG AGTATGCA--AGCCCGCTAGTGGCTAGTGGGAGATTCTCA
Gp-DNA         GG AAT TTG ACA AGG ACT ATG AGTATGTA--AATCCGCCCGTGG-------GAGATTTCCA
Ac-DNA         AG AAC CTG ACC ACT AGC ATG AGTGAGTAC-AACATCCCTGTGT-------GATGTAACTG
Ao-DNA         AA AAC CTG ACC AGG ACA ATG AGTGGGTGTTGGCTAGTTTGTTG-------GAGATTTTTA
Gd-PRO         R   N   L   T   R   T   M
Gp-PRO         R   N   L   T   R   T   M
Ac-PRO         K   N   L   T   T   S   M
Ao-PRO         K   N   L   T   R   T   M

Gd-DNA         ----ATTGCTAA-CA--ATT-GACA-GAG AAC ATC CAG GCA ACA TAT GAG TTG AGC CCT G
Gp-DNA         ----ATTGCTAA-CA--ATTTGGCA-GAG AAC ATA CAG GCA ACA TAT GAG CTG AGC CCT G
Ac-DNA         GAAGACCACTGA-CGCGGTATCACAAGAC AAC ATC CGG GCT ACA TAT AAG TTG ACC CCC G
Ao-DNA         TTGTCATGCTAAATACATTCTCGTTAGGA AAC ATC CAA GCT ACA TAC TCT CTG ACG GGT A
Gd-PRO                                        N   I   Q   A   T   Y   E   L   S   P
Gp-PRO                                        N   I   Q   A   T   Y   E   L   S   P
```

```
Ac-PRO                                                  N   I   R   A   T   Y   K   L   T   P
Ao-PRO                                                  N   I   Q   A   T   Y   S   L   T   G

Gd-DNA    CT GAC CGA ACC ATG CTT GTC ATG CCT CTT TTC CAC GTC CAC GGC CTT CTC GCC GGG CTC
Gp-DNA    CT GAC CGA ACC ATG CTC GTC ATG CCT CTT TTC CAC GTC CAC GGC CTT CTT GCC GGG TTC
Ac-DNA    AG GAT CGC ACC TAC TTG GTG ATG CCT CTA TTC CAC GTC CAC GGG CTG TTG GCC GGA TTC
Ao-DNA    AT GAC CGA ACT TAT CTT GTT ATG CCG CTC TTC CAC GTC CAC GGG CTT CTC GCC GCT TTC
Gd-PRO    A   D   R   T   M   L   V   M   P   L   F   H   V   H   G   L   L   A   G   L
Gp-PRO    A   D   R   T   M   L   V   M   P   L   F   H   V   H   G   L   L   A   G   F
Ac-PRO    E   D   R   T   Y   L   V   M   P   L   F   H   V   H   G   L   L   A   G   F
Ao-PRO    N   D   R   T   Y   L   V   M   P   L   F   H   V   H   G   L   L   A   A   F

Gd-DNA    CTC GCC CCT CTT CTT TCC GGA GGC TCT GTC GTT GTC CCT GCC AAG TTC AGC GCC ACT ACC
Gp-DNA    CTT GCC CCT CTA CTT TCC GGA GGG TCC GTC GTT GTC CCT GCC AAG TTC AGC GCC ACT ACT
Ac-DNA    CTC GCC CCC CTT TTG TCT GGA GGC TCC GTA ATT GTG CCT CCC AGA TTC TCC GCA TCG GAG
Ao-DNA    CTC GCC CCG TTA CAG TCT GGA GGG TCC GTC GTT GTG CCT CCC AAG TTC TCT GCT ACC GAT
Gd-PRO     L   A   P   L   L   S   G   G   S   V   V   V   P   A   K   F   S   A   T   T
Gp-PRO     L   A   P   L   L   S   G   G   S   V   V   V   P   A   K   F   S   A   T   T
Ac-PRO     L   A   P   L   L   S   G   G   S   V   I   V   P   P   R   F   S   A   S   E
Ao-PRO     L   A   P   L   Q   S   G   G   S   V   V   V   P   P   K   F   S   A   T   D

Gd-DNA    TTC TGG GAG GAT TTC ATC ACG CAT AAA GCC AAC TGG TAC ACT GCT GTG CCC ACC ATC CAC
Gp-DNA    TTC TGG GAA GAC TTT ATC AAT TAT AAA GCT AAC TGG TAC ACT GCT GTG CCC ACC ATC CAC
Ac-DNA    TTC TGG GCG GAT TTC GTT GGA TTC CAC GCG AAC TGG TAC ACA GCT GTG CCT ACG ATC CAT
Ao-DNA    TTC TGG TCG GAT TTC ATC ACG TAC AAG GCC AAC TGG TAT ACG GCG GTG CCG ACC ATA CAC
Gd-PRO     F   W   E   D   F   I   T   H   K   A   N   W   Y   T   A   V   P   T   I   H
Gp-PRO     F   W   E   D   F   I   N   Y   K   A   N   W   Y   T   A   V   P   T   I   H
Ac-PRO     F   W   A   D   F   V   G   F   H   A   N   W   Y   T   A   V   P   T   I   H
Ao-PRO     F   W   S   D   F   I   T   Y   K   A   N   W   Y   T   A   V   P   T   I   H

Gd-DNA    CAG ATT CTC CTC AGA AAC CCT CCT CCG TCC ACC AAG CCC AAC ATT CGA TTC ATT CGC TCT
Gp-DNA    CAG ATC CTT CTC AGA AAC CCA CCC CCG TCA GCC AAA CCT AAC ATT CGA TTT ATC CGG TCC
Ac-DNA    CAG ATC CTC CTT AAG ACA CCG CTG CCG AAC CCC ATT CCC AAG ATC CGG TTC ATT CGC TCA
Ao-DNA    CAG ATC CTA CTC AAG CAT CCA TTC CCA TCT CCC ATG CCA AAG ATC CGT TTC GTC CGC TCT
Gd-PRO     Q   I   L   L   R   N   P   P   P   S   T   K   P   N   I   R   F   I   R   S
Gp-PRO     Q   I   L   L   R   N   P   P   P   S   A   K   P   N   I   R   F   I   R   S
Ac-PRO     Q   I   L   L   K   T   P   L   P   N   P   I   P   K   I   R   F   I   R   S
Ao-PRO     Q   I   L   L   K   H   P   F   P   S   P   M   P   K   I   R   F   V   R   S
```

```
Gd-DNA    TGC TCG TCG CCT CTT TCG CCA ACA ACC TTC CAC CAG CTT GAG GAG CAG TAC AAT GCC CCT
Gp-DNA    TGC TCG TCT CCT TTA TCG CCA ACA ACC TTT TAC CAA CTC CAG GAG CAG TAC AAT GCT CCT
Ac-DNA    TGC TCA TCG CCG CTG TCG CCA AAG ACT TTC GAG GAT CTG GAA AAG ACG TTC AAG GCC CCC
Ao-DNA    TGC TCG TCA CCG CTG TCT CCC AAG ACA TTC CAC GAA ATA GAA CGT GCA TTC AAT GCT CCG
Gd-PRO     C   S   S   P   L   S   P   T   T   F   H   Q   L   E   E   Q   Y   N   A   P
Gp-PRO     C   S   S   P   L   S   P   T   T   F   Y   Q   L   Q   E   Q   Y   N   A   P
Ac-PRO     C   S   S   P   L   S   P   K   T   F   E   D   L   E   K   T   F   K   A   P
Ao-PRO     C   S   S   P   L   S   P   K   T   F   H   E   I   E   R   A   F   N   A   P

Gd-DNA    GTT CTC GAG GCA TAT GCC ATG ACT GAA GCC GCC CAT CAA ATG ACA TCC AGC CCT CTC CCA 484
Gp-DNA    GTC CTC GAG GCA TAT GCC ATG ACG GAA GCT GCC CAC CAA ATG ACC TCC AAC CCA CTT CCA 478
Ac-DNA    GTC CTG GAA GCG TAC GCG ATG ACC GAG GCT TCG CAC CAG ATG ACC AGC AAC CCT CTC CCG 486
Ao-DNA    GTT CTC GAA GCA TAT GCC ATG ACC GAA GCC TCA CAT CAA ATG ACA AGC AAC CCT CTA CCG 488
Gd-PRO     V   L   E   A   Y   A   M   T   E   A   A   H   Q   M   T   S   S   P   L   P
Gp-PRO     V   L   E   A   Y   A   M   T   E   A   A   H   Q   M   T   S   N   P   L   P
Ac-PRO     V   L   E   A   Y   A   M   T   E   A   S   H   Q   M   T   S   N   P   L   P
Ao-PRO     V   L   E   A   Y   A   M   T   E   A   S   H   Q   M   T   S   N   P   L   P
```

POB3 (FACT complex subunit): ≈400 bases aligned; The alignment starts with non-coding miscellaneous feature (Va = *Verticillium albo-atrum*, Gg = *Glomerella graminicola*)

```
Gd-DNA    --CAATTGGCGCA------CCCTCTAACCT-ATG-------TA CAG GAC --- TTC GAC CG
Gp-DNA    --CGATTGGCGCA------CCTTCTAACGT-ATG-------TA CAG GAC --- TTC GAC CG
Va-DNA    CGCGACACCCGCA------TCATCCAGCTCGACGGCTTCC-CG CAG GAC GAG TTC GAG CG
Gg-DNA    CCCAGTAAGTGGGGGAAGCCGAGCTAATTGAACACCCGGCGTG CAG GAC --- TAC GAG CG
Gd-PRO                                                 Q   D   -   F   D   R
Gp-PRO                                                 Q   D   -   F   D   R
Va-PRO                                                 Q   D   E   F   E   R
Gg-PRO                                                 Q   D   -   Y   E   R

Gd-DNA    C CTG ACG AAA GTA TTC AAG AAC TGG TAC AGC ACG AAC CTC GAG ACG AAG GAG CAC GCA TT
Gp-DNA    C CTG ACG AAA GTG TTC AAG AAC TGG TAC AGC ACG AAC CTC GAG ACG AAG GAA CAC GCA TT
Va-DNA    C CTG TCG AAG CTC TTC AAA AAC TGG TAC AGC ACG ACC CTC GAG AAC AAG GAG CAC GCC CT
Gg-DNA    C CTC GCA AAG ATT TTC AAG AAT TGG TAC AGC ACC GCC CTC GAG AAT AAG GAG CAC GCC CT
Gd-PRO       L   T   K   V   F   K   N   W   Y   S   T   N   L   E   T   K   E   H   A   L
```

```
Gp-PRO       L   T   K   V   F   K   N   W   Y   S   T   N   L   E   T   K   E   H   A   L
Va-PRO       L   S   K   L   F   K   N   W   Y   S   T   T   L   E   N   K   E   H   A   L
Gg-PRO       L   A   K   I   F   K   N   W   Y   S   T   A   L   E   N   K   E   H   A   L

Gd-DNA   A CGA GGA TGG AAC TGG GGC AAG GCG GAA TTC GGA AAG GCG GAA TTG GCA TTC AAC GTG CA
Gp-DNA   A CGA GGA TGG AAC TGG GGG AAA GCG GAG TTC GGA AAG GCG GAG CTG GCA TTC AAT GTG CA
Va-DNA   G CGC GGC TGG AAC TGG GGC AAG GCG GAG TTC GGC AAG GCT GAG CTG TCC TTC AAC GTG CA
Gg-DNA   G AGA GGA TGG AAC TGG GGC AAG GCC GAA TTC GGA AAG GCC GAG CTT TCC TTC AAC GTC CA
Gd-PRO       R   G   W   N   W   G   K   A   E   F   G   K   A   E   L   A   F   N   V   Q
Gp-PRO       R   G   W   N   W   G   K   A   E   F   G   K   A   E   L   A   F   N   V   Q
Va-PRO       R   G   W   N   W   G   K   A   E   F   G   K   A   E   L   S   F   N   V   Q
Gg-PRO       R   G   W   N   W   G   K   A   E   F   G   K   A   E   L   S   F   N   V   Q

Gd-DNA   G AAC AGA CCT GCG TTT GAG ATC CCC TAC TCG GAG ATC TCG AAT ACG AAT TTG GCT GGG AA
Gp-DNA   G AAC AGA CCT GCG TTT GAA ATT CCT TAC TCG GAG ATC TCG AAT ACG AAT TTG GCT GGA AA
Va-DNA   G AAT CGT CCG GCC TTT GAG GTG CCC TAC TCG GAA ATC TCC AAC ACC AAC CTC GCT GGC CG
Gg-DNA   G AAC CGA CCT GCT TTT GAA ATC CCT TAC TCC GAG ATC TCA AAT ACC AAC CTG GCA GGT CG
Gd-PRO       N   R   P   A   F   E   I   P   Y   S   E   I   S   N   T   N   L   A   G   K
Gp-PRO       N   R   P   A   F   E   I   P   Y   S   E   I   S   N   T   N   L   A   G   K
Va-PRO       N   R   P   A   F   E   V   P   Y   S   E   I   S   N   T   N   L   A   G   R
Gg-PRO       N   R   P   A   F   E   I   P   Y   S   E   I   S   N   T   N   L   A   G   R

Gd-DNA   G AAC GAG GTG GCG GTT GAG TTC TCG TTG CCG GCT GGT --- GGC GAT GAG GGC GCA AAC G
Gp-DNA   G AAC GAG GTG GCA GTC GAG TTT TCG TTG CCG GCG GGT --- GGC GAC GAG GGC GCA AAC G
Va-DNA   C AAC GAG GTC GCC GTC GAG TTT GCC GCC CCC ACC GAC GAG AAC GAC ACA GGC ACC AAC G
Gg-DNA   C AAC GAA GTC GCC GTC GAA TTC TCT GCC CCG ACG GAC CAG AAC GAT ACC GGC ACG AAT G
Gd-PRO       N   E   V   A   V   E   F   S   L   P   A   G   -   G   D   E   G   A   N
Gp-PRO       N   E   V   A   V   E   F   S   L   P   A   G   -   G   D   E   G   A   N
Va-PRO       N   E   V   A   V   E   F   A   A   P   T   D   E   N   D   T   G   T   N
Gg-PRO       N   E   V   A   V   E   F   S   A   P   T   D   Q   N   D   T   G   T   N
Gd-DNA   GA AGC TTG GGT GGT GCG AAG GGG AAG GGG AAG AAG GCT GGC GCG GGG AAG GAT CAG TTG G 359
Gp-DNA   GA AGC TTG GGC GGT GCG AAG GGG AAG GGC AAG AAG GCC GGC GCA GGG AAG GAT CAA TTA G 393
Va-DNA   GG ACT CTC GAC GGT GCG CGT GGC AAG GGC AAG AAG GCG GGT GCT GGC AAG GAC CAG CTC G 459
Gg-DNA   GC CAC TTG GGC GGC GCG CGA GGA AAG GGC AAG AAG GCC GGA GCC GGC AAA GAT CAG CTC G 465
Gd-PRO   G   S   L   G   G   A   K   G   K   G   K   K   A   G   A   G   K   D   Q   L
Gp-PRO   G   S   L   G   G   A   K   G   K   G   K   K   A   G   A   G   K   D   Q   L
Va-PRO   G   T   L   D   G   A   R   G   K   G   K   K   A   G   A   G   K   D   Q   L
```

```
Gg-PRO          G   H   L   G   G   A   R   G   K   G   K   K   A   G   A   G   K   D   Q   L
```

SRP72 (Signal recognition particle protein 72): ~400 bases aligned; Region shown include 5′ UTR and 5′ region of the protein (Ss = *Sclerotinia sclerotiorum*, Ad = *Ajellomyces dermatiditis*)

```
Gd-DNA          CTAAAATATACATATACATGGAATGAAACATATGCCGCGCATATAATACATATGATATCAGCTTACTCAAGTGCGCCCAAT
Ss-DNA          GCCGCGCTAGCTCAGCAGTTGATCGCTTTTGACACCATCTCCATATCGCATGGCTACAGTAAATAGGAACCCATCGCCCTA
Gp-DNA          GGAGATATACATACACATGGACTGAAATATATATGGCCTCGCATATAATCAACATATCAGTTTACTCAAGTGCGCCTAATA
Ad-DNA          TTATTCGCCAACCTTCCTTCTTTTTTATTTCTTTAAACAATCCTTTTCGGGAGCCAGTGCGGCGCCTGAACAGCCCTTGAA

Gd-DNA          CTC GCT AGC ATG GCG TCC AAC CCC ACC GCT ACG TTG AGC AAA CTA CTC GGC TCA GCA ACT A
Ss-DNA          GAG TTT AAA ATG GCC --- GAT CAA ACA GCA ACG CTC AGC GCC CTC CTT CGT AAT TCT TCA A
Gp-DNA          CTC GCT AGC ATG GCG TCC AAC CCC ACC GCT ACC TTG AGC AAA CTC CTC GGC TCA GCA ACT A
Ad-DNA          CTT CTC CGT ATG GCG --- --- --- ACC CAG AGC ATC AGT GCG TTG GTT CAG CGA AGC ACA A
Gd-PRO                       M   A   S   N   P   T   A   T   L   S   K   L   L   G   S   A   T
Ss-PRO                       M   A   -   D   Q   T   A   T   L   S   A   L   L   R   N   S   S
Gp-PRO                       M   A   S   N   P   T   A   T   L   S   K   L   L   G   S   A   T
Ad-PRO                       M   A   -   -   -   T   Q   S   I   S   A   L   V   Q   R   S   T

Gd-DNA          TG GAG GAC CAT GAG GAG ATT CTG AGA GCC GCC AAT GCC GTT CTC AAG AAG TCG AAA ACA A
Ss-DNA          TT ACA GAC CAT GAA GAG ATC CTC AAA GCT ACA AAT GAC GTA TTA AAG ACT TCT AAA CTC G
Gp-DNA          TC GAT GAC CAC GAG GAG GTT CTG AGA GCC GCC AAT GCT ATT CTC AAG AAG TCG AAA ACA G
Ad-DNA          TC GAC GAC CAT GAA GAA GTC ATC AAA GCT TGC AAT GCG GTA CTG AAA ACG TCC AAA AAC G
Gd-PRO          M   E   D   H   E   E   I   L   R   A   A   N   A   V   L   K   K   S   K   T
Ss-PRO          I   T   D   H   E   E   I   L   K   A   T   N   D   V   L   K   T   S   K   L
Gp-PRO          I   D   D   H   E   E   V   L   R   A   A   N   A   I   L   K   K   S   K   T
Ad-PRO          I   D   D   H   E   E   V   I   K   A   C   N   A   V   L   K   T   S   K   N

Gd-DNA          AC CAG GAT GCG CTG CGT ACT CGC GTT ATT GCA CTC CTC AAG CTA GAC CGC TAC GCA GAT G
Ss-DNA          AC CCC GAT GCC TTG CAT ACC CGC GCT ATT GCT CTC CTC AAA CTT GAT CGA TTC GAC GAT G
Gp-DNA          AC CAG GAT GCG CTA CGT ACT CGT GTC ATT GCG CTG CTT AAG CTA GAC CGC TAC GCA GAT G
Ad-DNA          AT CTC AAT GCC CTC CAT GTA AAA ACT GTT GCC CTG GTC AAA TTG GAC CGT TTC GAA GAT G
Gd-PRO          N   Q   D   A   L   R   T   R   V   I   A   L   L   K   L   D   R   Y   A   D
Ss-PRO          D   P   D   A   L   H   T   R   A   I   A   L   L   K   L   D   R   F   D   D
Gp-PRO          D   Q   D   A   L   R   T   R   V   I   A   L   L   K   L   D   R   Y   A   D
Ad-PRO          D   L   N   A   L   H   V   K   T   V   A   L   V   K   L   D   R   F   E   D
```

```
Gd-DNA    CC CTC CGC GCC CTC GAC GAC GGG GGC GAA GCT CTC AGC GAA AGC TGC CAC GTT
Ss-DNA    CA CTA AAA GCC CTC GAC GAA GGT GGA GAT AAA TTG GCT TCT CAA TGT ATT CTC
Gp-DNA    CT CTC CGC GCC CTC GAC GAC GGG GGA GAT GCT CTC AGC GAA ATC TGC CAC GTT
Ad-DNA    CG ATT CGA GTG ATC GAG GAT GGC GGG GAT GCG CTG AAA AAG AAA GTC CCT CTG
Gd-PRO    A  L   R   A   L   D   D   G   G   E   A   L   S   E   S   C   H   V
Ss-PRO    A  L   K   A   L   D   E   G   G   D   K   L   A   S   Q   C   I   L
Gp-PRO    A  L   R   A   L   D   D   G   G   D   A   L   S   E   I   C   H   V
Ad-PRO    A  I   R   V   I   E   D   G   G   D   A   L   K   K   K   V   P   L

Gd-DNA    GAG AAG TCG TAC GCC CTT TAC AAA ACA GGC CAG CTC GAG GCC GCA CAG AAG ATT TTC G
Ss-DNA    GAA CGC GCT TAT GCT CTC TAC AAG ACA GGG AAA CTG GCA GAT GCG GCG AAA GTA TGT G
Gp-DNA    GAG AAA TCG TAC GCG CTT TAC AAA ACA GGC CAA CTC GAG GCC GCA CAG AAA ATT TTC G
Ad-DNA    GAA TGG TCT TAC GCT TTA TAT AAG GTC GGG CAA TTA GAA GAT GCG ATA AAA CTG GCC G
Gd-PRO     E   K   S   Y   A   L   Y   K   T   G   Q   L   E   A   A   Q   K   I   F
Ss-PRO     E   R   A   Y   A   L   Y   K   T   G   K   L   A   D   A   A   K   V   C
Gp-PRO     E   K   S   Y   A   L   Y   K   T   G   Q   L   E   A   A   Q   K   I   F
Ad-PRO     E   W   S   Y   A   L   Y   K   V   G   Q   L   E   D   A   I   K   L   A
```

VPS13 (Vacuolar protein sorting-associated protein): ~545 bases aligned (Ss = *Sclerotinia sclerotiorum*, Bf = *Botryotinia fuckeliana*)

```
Gd-DNA    GG ATC CCA GAT AGT GGT GGA GCG TTT AGA CTC ACG ATT TAC AGT CCT TAC ATC ATT TTG A
Gp-DNA    GA ATC CCA GAC GGC GGT GGA GCG TTT AGA CTC ACA ATT TAC AGT CCT TAT GTC ATT TTG A
Ss-DNA    AG ATT CCA GAT AGT GGA GGA GCA TTT AGA GTA ACT GTC TAC AGT CCT TAC GTT ATC CTA A
Bf-DNA    AG ATT CCA GAT AGT GGT GGA GCT TTT AGA GTC ACC GTA TAC AGT CCT TAT GTA ATC CTA A
Gd-PRO        I   P   D   S   G   G   A   F   R   L   T   I   Y   S   P   Y   I   I   L
Gp-PRO        I   P   D   G   G   G   A   F   R   L   T   I   Y   S   P   Y   V   I   L
Ss-PRO        I   P   D   S   G   G   A   F   R   V   T   V   Y   S   P   Y   V   I   L
Bf-PRO        I   P   D   S   G   G   A   F   R   V   T   V   Y   S   P   Y   V   I   L

Gd-DNA    AT AAA ACT GGC CTG GAT ATC AAT ATC AAG GCC AAG TCG TTA CTG CAG CAA GCA CGG ACG G
Gp-DNA    AT AAA ACA GGC CTG GAT ATC AAC ATC AAG GCG AAG TCG TTA CTG CAG CAA GCG CGG ACT G
Ss-DNA    AT AAA ACC GGA CTG GAG ATC AAC ATC AAA CAG AAG TCA TTG TTG CAG CAA GCA AAG ACG G
Bf-DNA    AT AAG ACT GGC CTA GAG ATC AAC ATC AAA CAA AAA TCG CTA TTG CAG CAA GCA AAG ACG G
Gd-PRO    N  K   T   G   L   D   I   N   I   K   A   K   S   L   L   Q   Q   A   R   T
Gp-PRO    N  K   T   G   L   D   I   N   I   K   A   K   S   L   L   Q   Q   A   R   T
```

Ss-PRO	N K T G L E I N I K Q K S L L Q Q A K T
Bf-PRO	N K T G L E I N I K Q K S L L Q Q A K T

Gd-DNA	CG GCT GGC CAG AAG GTA GTG CGC GAT CTC CTT GGG GAT GAC GAA CAG AAG GCG CTG CCT C
Gp-DNA	CA GCT GGA CAG AAG GTC GTG CGC GAT CTC CTC AGC GAT GAC GAG CAG AAG GCG CTG CCT T
Ss-DNA	CG GCC GGT CAA GGG TTT CGT ACA GAT TCG GCT GAT TCA GAA CGG CGT AAG GCA CTA CCT T
Bf-DNA	CA GCA GGT CAA GGG TTC CAT ACT GAC TCA GTC GAT TCT GAA CGG CGC AAA GCA CTA CCA T
Gd-PRO	A A G Q K V V R D L L G D D E Q K A L P
Gp-PRO	A A G Q K V V R D L L S D D E Q K A L P
Ss-PRO	A A G Q G F R T D S A D S E R R K A L P
Bf-PRO	A A G Q G F H T D S V D S E R R K A L P

Gd-DNA	TG ATG TTT GCG TTC AGT GGC GAC GAC CAG CGC AAC CGC GTC ATC CTC AAA GTC GGC GAG T
Gp-DNA	TG ATG TTT GCG TTC AGT GGC GAC GAC CAG CGT AAC CGC GTC ATC CTC AAA GTT GGC GAG T
Ss-DNA	AT ATG TTC GCA TAT GGC GGA GAT GAT CAA AGG AAT CGT GCT ATA TTG AAG GTT GGC GAC T
Bf-DNA	AC ATG TTC GCG TAT GGC GGA GAT GAT CAA CGA AAT CGT GCT TTA ATG AAG GTC GGT GAC T
Gd-PRO	L M F A F S G D D Q R N R V I L K V G E
Gp-PRO	L M F A F S G D D Q R N R V I L K V G E
Ss-PRO	Y M F A Y G G D D Q R N R A I L K V G D
Bf-PRO	Y M F A Y G G D D Q R N R A L M K V G D

Gd-DNA	CG AAC TGG AGT AAA CCG CAG AGT TTC GAC GCC ATT GGC AGC ACA ATC GAT GTG GTT CTA C
Gp-DNA	CG AAC TGG AGT AAA CCG CAG AGT TTC GAC GCC ATT GGC AGC ACG ATT GAT GTT GTT CTA C
Ss-DNA	CG AAT TGG AGT AAA CCA CAA AGT CTG GAT GCC ATC GGT AGT AAT GTC GAT GTT GTT TTA T
Bf-DNA	CC AAC TGG AGT AAA CCG CAA AGT TTG GAT GCT ATC GGC AGC AAT GTA GAT GTC ATA TTG C
Gd-PRO	S N W S K P Q S F D A I G S T I D V V L
Gp-PRO	S N W S K P Q S F D A I G S T I D V V L
Ss-PRO	S N W S K P Q S L D A I G S N V D V V L
Bf-PRO	S N W S K P Q S L D A I G S N V D V I L

Gd-DNA	CG TCA GCA ACT CAG AAT ACT GAG ATC CAT GTT GGA ATC AGC ATC GAG AAT GGA GAC GGG A
Gp-DNA	CC TCG GCA ACT CAG AAT ACC GAG ATA CAC GTT GGA ATC AGT ATC GAG AAC GGG GAC GGG A
Ss-DNA	CT TCT TCT AAA AAC AAC ACC GAG ATA CAC GTG GGC ATC AAT GTT GAA TCA GGA GAG GGC A
Bf-DNA	CT TCC TCA AAG AAC AAT ACC GAA ATT CAT GTA GGT ATC AGT GTC GAA GCA GGA GAG GGT A
Gd-PRO	P S A T Q N T E I H V G I S I E N G D G
Gp-PRO	P S A T Q N T E I H V G I S I E N G D G
Ss-PRO	S S S K N N T E I H V G I N V E S G E G

```
Bf-PRO     P  S  S  K  N  N  T  E  I  H  V  G  I  S  V  E  A  G  E  G

Gd-DNA     AA TAC AAG ATG ACC AAG GTC GTT ACG CTT GCG CCG AGA TTT GTG CTG AAG AAT CGC ATG A
Gp-DNA     AA TAC AAG ATG ACC AAG GTC GTT ACG CTT GCA CCG AGA TTT GTG CTG AAG AAT CGC ATG A
Ss-DNA     AA TAC AAA ATG ACG AAG GTA GTC ACT CTT GCT CCC CGC TTC GTT CTG AAG AAT CAG ATG A
Bf-DNA     AA TAT AAG ATG ACG AAG GTT GTC ACA CTT GCT CCT CGC TTT GTT TTA AAG AAT CAA ATG A
Gd-PRO     K  Y  K  M  T  K  V  V  T  L  A  P  R  F  V  L  K  N  R  M
Gp-PRO     K  Y  K  M  T  K  V  V  T  L  A  P  R  F  V  L  K  N  R  M
Ss-PRO     K  Y  K  M  T  K  V  V  T  L  A  P  R  F  V  L  K  N  Q  M
Bf-PRO     K  Y  K  M  T  K  V  V  T  L  A  P  R  F  V  L  K  N  Q  M


Gd-DNA     GC GAG GAA ATC AGC GCC AGG GAA CCT GGA TCG TCG GAG CTT ATG ACT TTG AAG CGG GGA G
Gp-DNA     GT GAG GAA ATC AGC GCC AGG GAA CCT GGA TCG TCG GAG CTC ATG ACT TTG AAG CCG GGA G
Ss-DNA     AC GAG GAA ATA AAT GTC AGG GAA CCC GGG TCG TCT GAA CTC TGG ACT TTG AAG CCA CAG G
Bf-DNA     GC GAA GAA CTA AAC GTT AGA GAG CCT GGG TCA TCT GAA CTT TGG ACT TTG AAG CCA CAA G
Gd-PRO     S  E  E  I  S  A  R  E  P  G  S  S  E  L  M  T  L  K  R  G
Gp-PRO     S  E  E  I  S  A  R  E  P  G  S  S  E  L  M  T  L  K  P  G
Ss-PRO     N  E  E  I  N  V  R  E  P  G  S  S  E  L  W  T  L  K  P  Q
Bf-PRO     S  E  E  L  N  V  R  E  P  G  S  S  E  L  W  T  L  K  P  Q

Gd-DNA     AA CTG AAG GCT CTT CAC TTC CTT CAA AAG TCG GCG GTT AAG CAA CTG TGC CTT TGC TTC C
Gp-DNA     AA CTG AAA GCT CTT CAC TTC CTT CAA AAG TCG GCG GTG AAG CAA CTG TGC CTT TGC TTC C
Ss-DNA     CC CTA GAA CCT CTT CAT TTC CTC CAG AAG AGT CCC GTC AAG CAA CTT ACG CTA TGC TTC C
Bf-DNA     CT TTA GAG CCT CTT CAT TTC CTT CAG AAG AGT CAT GTA AAA CAA CTC ACG ATG TGC TTC C
Gd-PRO     E  L  K  A  L  H  F  L  Q  K  S  A  V  K  Q  L  C  L  C  F
Gp-PRO     E  L  K  A  L  H  F  L  Q  K  S  A  V  K  Q  L  C  L  C  F
Ss-PRO     A  L  E  P  L  H  F  L  Q  K  S  P  V  K  Q  L  T  L  C  F
Bf-PRO     A  L  E  P  L  H  F  L  Q  K  S  H  V  K  Q  L  T  M  C  F

Gd-DNA     CC GGC
Gp-DNA     CG GGC
Ss-DNA     CT GGA
Bf-DNA     CA GGA
Gd-PRO     P  G
Gp-PRO     P  G
Ss-PRO     P  G
Bf-PRO     P  G
```
